# Mindfulness, perceived stress, and subjective well-being: a correlational study in primary care health professionals

**DOI:** 10.1186/s12906-015-0823-0

**Published:** 2015-09-02

**Authors:** Ana C. M. Atanes, Solange Andreoni, Marcio S. Hirayama, Jesús Montero-Marin, Viviam V. Barros, Telmo M. Ronzani, Eliza H. Kozasa, Joaquim Soler, Ausiàs Cebolla, Javier Garcia-Campayo, Marcelo M. P. Demarzo

**Affiliations:** Mente Aberta – Brazilian Center for Mindfulness and Health Promotion, Department of Preventive Medicine, Escola Paulista de Medicina, Universidade Federal de Sao Paulo, UNIFESP, Rua Botucatu, 740, 04023-900 Sao Paulo, SP Brazil; University of Zaragoza, Calle Pedro Cerbuna, 12, 500009 Zaragoza, Spain; Federal University of Juiz de Fora, Campus Universitário, Martelos, 36036-900 Juiz de Fora, MG Brazil; Department of Psychobiology, São Paulo Medical School, Federal University of Sao Paulo, UNIFESP, Rua Botucatu, 740, 04023-900 Sao Paulo, SP Brazil; Instituto do Cérebro, Inst. Israelita de Ensino e Pesquisa Albert Einstein, Av. Albert Einstein, 627/701, CEP, 05601-901 São Paulo, SP Brazil; Department of Psychiatry, Hospital de la Santa Creu i Sant Pau, Barcelona, Spain; Universitat Autònoma de Barcelona (UAB), Centro de Investigación Biomédica en Red de Salud Mental (CIBERSAM), Institut d’Investigació Biomédica- Sant Pau (IIB-SANT PAU), Barcelona, Spain; Departament de Psicologia Bàsica, Clínica i Psicobiologia, Universitat Jaume I, Castelló, Spain; CIBER Fisiopatologia de la Obesidad y la Nutrición (CIBERobn), Santiago de Compostela, Spain

**Keywords:** Mindfulness, Perceived stress, Subjective well-being, Primary health care professionals, Health services research

## Abstract

**Background:**

Primary health care professionals (PHPs) usually report high levels of distress and burnout symptoms related to job strain. Mindfulness, defined as non-judgmental-present-moment awareness, seems to be a moderator in the causal association between life stressors and well-being. This study aimed to verify correlations among self-reported mindfulness, perceived stress (PS), and subjective well-being (SW) in Brazilian PHPs.

**Methods:**

We performed a correlational cross-sectional study in a purposive sample of Brazilian PHPs (physicians, nurses, nursing assistants, and community health workers), working in community-oriented primary care programs (known locally as “Family Health Programs”). We used validated self-reporting instruments: the Mindful Attention Awareness Scale (MAAS), the Perceived Stress Scale (PSS), and the Subjective Well-being Scale (SWS). We performed a multivariate analysis of variance (MANOVA), through regression coefficients (beta) in relation to the professional category (nursing assistant), in addition to the length of time in the same job (under than 6 months) that had indicated the lowest level of PS.

**Results:**

Participants (*n* = 450) comprised community health workers (65.8 %), nursing assistants (18 %), registered nurses (10.0 %), and doctors (family physicians) (6.0 %); 94 % were female and 83.1 % had worked in the same position for more than one year. MANOVA regression analysis showed differences across professional categories and length of time in the same job position in relation to mindfulness, PS, and SW. Nurses demonstrated lower levels of mindfulness, higher PS, and SW negative affect, as well as lower SW positive affect. Being at work for 1 year or longer showed a clear association with higher PS and lower SW positive affect, and no significance with mindfulness levels. Pearson’s coefficient values indicated strong negative correlations between mindfulness and PS, and medium correlations between mindfulness and SW.

**Conclusion:**

In this study, there were clear correlations between mindfulness, PS, and SW across different primary care professional categories and time in the same job position, suggesting specific vulnerabilities that should be addressed through the development of staff awareness, stress prevention, and well-being interventions.

## Background

There is a growing interest in the associations between awareness and the well-being of health care professionals [1], as a result of positive research evidence on mindfulness in this population [2]. Mindfulness involves the self-regulation of attention to the experience of the present moment and decentered, non-judgmental awareness, referring to openness to one’s internal experiences and external events [3, 4]. Mindfulness has its roots in Buddhist philosophy, but its current construct goes beyond religious concepts [4] and can be improved by attention training and meditation [4, 5].

Trait mindfulness has proved to be a protective characteristic, showing negative correlations with stress and positive correlations with well-being [6]. Stress and burnout are prevalent amongst PHPs in Brazil, and are considered a problem affecting the health of PHPs and provision of high standard health services [7, 8], suggesting the need for further studies on this population.

From a sample of professionals from the Family Health Strategy (FHS) of the Unified Health System of Brazil, 62 % presented high levels of perceived stress (psychological symptoms in 48 %, physical in 39 % and both symptoms in 13 %) [9]. Research shows that these type of professionals report lack of adequate training, work overload, poor work conditions, and feelings of professional helplessness and frustration [8, 10, 11].

The health system works by dividing the provision of care into metropolitan areas (health districts), whereas the FHS model is based on the “team/community/family and team/team” “bonding” relationship [11]. Among many difficulties the strategy faces is the non-comprehension of some workers and health administrators about its purpose [10]. Furthermore, evidence also indicates that demographic aspects can be a risk factor for burnout in this population, with men showing twice as much propensity to develop exhaustion. Age was also another factor [8], specifically in PHPs aged 29 years or less [12]. Those working at FHS as their first employment experience showed a four times greater likelihood of displaying burnout [13]. Finally, Leonelli and colleagues measured perceived stress in a sample of FHS professionals, showing differences in (PS levels per PHP category and length of time working in the job. They observed that one year or more in the same position predicted high levels of PS [14].

Evidence taken from samples of PHPs worldwide has shown, however, that an 8-week Mindfulness-Based Stress Reduction (MBSR) [3] intervention, based on exercises of meditation, attention control to experience without elaboration, affective attitude of openness, and other coping strategies, decreased PS, distress, and burnout, and increased self-compassion and subjective well-being - satisfaction with life (SWS_SL) [15]. Irving, Dobkin and Park’s review on MBSR and health professionals also showed significant evidence of better physical and mental health after completion of MBSR programs [2].

Among validated and well-established mindfulness instruments measuring trait mindfulness, the Mindful Attention Awareness Scale (MAAS) [6] was validated in Brazil on the general population, and correlated negatively with PS and SW negative affect and positively with SW positive dimensions [16]. Similar associations were expected between these variables in Brazilian PHPs. Considering the aforementioned, we also hypothesized associations between length of service and professional category with states of well-being, because of the possible accumulative effects of work conditions over time, and possible differential role conflicts and ambiguities within the FHS model.

Therefore, in order to start exploring trait mindfulness and to direct future mindfulness-based interventions (MBIs) on professionals in the FHS, the main goal of the present study was to explore these associations across different professional categories, taking into consideration the length of time in the same job.

## Methods

### Design

This was a cross-sectional correlational study. Subjects (*N* = 570) comprised the total population working in all the community-oriented primary care facilities of Sapopemba, one of the largest health districts in the city of Sao Paulo, Brazil. We chose this particular area for its characteristics of including a large sample of PHPs under the same type of employment contract and similar roles across different health facilities. Eligibility criteria included all PHPs who were combined in core teams: family physicians (FPs), registered nurses (RNs), nursing assistants (NAs), and community health workers (CHWs); on permanent employment contracts; not on annual leave or absent due to health problems; and voluntary participation.

### Procedure and ethics

We collected data between October, 2011, and February, 2012, and invited participants via local managers, phone calls, posters, and other printed materials, and visits to FHS facilities. The questionnaires were answered collectively, during working hours, in accordance with every FHS timetable, for the purpose of causing minimum disturbance to the service, and with flexibility to fit working patterns. Participants signed an informed consent form. The Ethical Committee of the Federal University of Sao Paulo (UNIFESP) approved the study protocol.

### Measures

We used instruments validated for the Brazilian Portuguese language and population; all described below:

I. General socio-demographic questionnaire for sample characterization was applied to determine sex; age; professional category; type of employment/type of contract; educational level; training for FHS job; time working in the job; family income; relationship status.
II. The Mindful Attention Awareness Scale (MAAS) [6] is a unidimensional, 15-item instrument, rated on a 6-point Likert scale (almost always to almost never). Higher scores indicate increased mindfulness, related to the level of awareness and attention to present events. An example item is, “I find myself preoccupied with the future or the past”. The Brazilian MAAS [16] showed adequate internal consistency (α = 0.83).
III. Perceived Stress Scale (PSS) [17] is a 14-item instrument rated on a 4-point Likert scale (almost never to always), which evaluates the perception of stressful events. An example item is, “In the last month, how often have you felt nervous and stressed?” We added the scores of all questions to calculate total scores, inversely calculating Items with positive connotation against the stress (0 = 4, 1 = 3, 2 = 2, 3 = 1, 4 = 0). The PSS showed adequate internal consistency (α = 0.82).
IV. Subjective Well-being Scale (SWS) is a 62-item instrument (developed in Brazil) divided into 3 subscales, all showing good internal consistency (α): positive affect (SWS_PA) (α = 0.95), negative affect (SWS_NA) (α = 0.95), and satisfaction with life (SWS_SL) (α = 0.90) [18]. The affects subscales are a 5-point Likert-type scale (not at all to extremely), consisting of 22 items of positive affect (e.g., “happy”) and 26 items of negative affect (e.g., “sad”). The third subscale is a 5-point Likert-type scale (I totally disagree to I totally agree), consisting of 14 items of satisfaction with life (e.g., “I am unsatisfied with my life”). Total scores are calculated by the sum of all items and reversed items were recoded (1 = 5, 2 = 4, 3 = 3, 4 = 2, 5 = 1).

To simplify comparability between the instruments, we transformed all scores into a one-hundred-point scale (0–100 points, with 100 meaning the highest level of mindfulness, PSS, and SWS).

### Data analysis

Descriptive analysis was reported by means and standard deviation (continuous variables); and absolute and relative frequencies (categorical variables). To verify the internal consistency of MAAS on this particular professional population we used Cronbach’s alpha coefficient, the item-rest correlation (correlation between an item and the total score formed by all the other items), and the alpha score without each item).

We performed a multivariate analysis of variance (MANOVA) through regression coefficients (β) in relation to the professional category (using nurse assistant as reference), as well as the length of time in the same job position, (using under 6 months as reference), which had indicated the lowest level of PS (p < 0.05), for every dependent variable. We verified relational concepts by calculating Pearson’s correlation coefficients between MAAS and PSS and SWS, also controlling by professional category and length of time in the same job. Data were analyzed using the SPSS 15 package.

## Results

### Characteristics of participants

We evaluated the 450 PHPs who agreed to participate in the study. Participants consisted of 65.8 % CHWs (*n* = 296), 18 % NAs (*n* = 82), 10.0 % RNs (*n* = 45) and 6.0 % FPs (*n* = 27), the mean age was 36.7 (SD = 9.1), 94 % were female, and 83.1 % had work period over 1 year (Table 1). Descriptives and internal consistency of the questionnaires can be seen in Table 2. Total Cronbach’s alphas of the scales showed good internal consistency, with all of them ≥ 0.85.

**Table 1:** Characteristics of the participants

Characteristic	Number	Percent
Total	450	100
Professional		
Community health worker	296	65.8
Nursing assistant	82	18.2
Registered nurse	45	10.0
Family physician	27	6.0
Sex		
Female	423	94.0
Male	27	6.0
Length of time in job		
Under 6 months	36	8.0
6 months – 1 year	39	8.7
1 – 2 years	110	24.4
2 – 5 years	121	26.9
Over 5 years	143	31.8
Did not answer	1	0.2

**Table 2:** Cronbach’s alpha and descriptives for the PSS, SWS_PA, SWS_NA and SWS_LS

	n	Mean	SD	Min – Max	Q_25_	Median	Q_75_	α
PSS	450	42.2	13.9	7.1 - 87.5	32.1	42.6	51.8	0.850
SWS_PA	448	57.7	17.3	0 - 98.8	46.4	58.3	70.2	0.951
SWS_NA	449	26.9	17.8	0 - 89.4	13.5	24.4	37.0	0.952
SWS_SL	448	64.5	17.4	16.1 - 100	52.5	66.1	76.8	0.897
MAAS	450	68.1	17.5	6,7-100	56.0	69.3	82.7	0.884

*PSS* Perceived Stress Scale, *SWS_PA* Subjective Well-being Scale - Positive affect, *SWS_NA* Subjective Well-being Scale - Negative affect, *SWS_SL* Subjective Well-being Scale - Satisfaction with life, *MAAS* The Mindful Attention Awareness Scale

### Professional categories and length of service

As seen in Table 3, FPs showed the lowest mindfulness scores (β = −20.1, *p* < 0.001), followed by RNs (β = −9.9, *p* = 0.002). No significant differences were found in CHWs (β = −3.2, *p* = 0.138) or in being in the job for 1 year or longer (β = −2.9, *p* = 0.196).

**Table 3:** Manova regression in relation to health professional category and length of service

Dependent variable	Independent variable^a^	Β	95 % CI	p
Perceived stress scale	CHW	4.0	[0.6; 7.4]	0.022
	RN	5.5	[0.5; 10.5]	0.032
	FP	3.4	[−2.8; 9.7]	0.275
	1 year or more in the job	4.8	[1.3; 8.3]	0.008
SWS – Positive affect	CHW	−6.5	[−10.6; −2.3]	0.002
	RN	−6.5	[−12.6; 0.3]	0.039
	FP	−1.5	[−9.0; 6.1]	0.705
	1 year or more in the job	−9.0	[−13.3; −4.7]	< 0.001
SWS – Negative affect	CHW	6.3	[2.0; 10.6]	0.004
	RN	10.2	[3.8; 16.6]	0.002
	FP	7.9	[0.01; 15.8]	0.050
	1 year or more in the job	5.3	[0.8; 9.8]	0.020
SWS – Satisfaction with life	CHW	−7.9	[−12.0; −3.7]	< 0.001
	RN	0.4	[−5.8; 6.6]	0.899
	FP	0.7	[−6.9; 8.4]	0.848
	1 year or more in the job	−5.0	[−9.3; −0.7]	0.023
Mindful Attention Awareness Scale	CHW	−3.2	[−7.3; 1.0]	0.138
	RN	−9.9	[−16.1; −3.7]	0.002
	FP	−20.1	[−27.8; −12.5]	< 0.001
	1 year or more in the job	−2.9	[−7.2; 1.5]	0.196

*SWS* Subjective Well-being Scale, *CHW* Community Health Worker, *RN* Registered Nurse, *FP* Family Physician

^a^Reference category: Nursing Assistant and under 6 months in the job

Higher PS scores were observed in RNs (β = 5.5, *p* = 0.032), followed by CHWs (β = 4.0, *p* = 0.022), and FPs (β = 3.4, *p* = 0.275). Lower mean scores in positive affect were observed in CHWs (β = −6.5, *p* = 0.02) and RNs (β = −6.5, *p* = 0.039). No significant difference was found between GPs and NAs (β = −1.5, *p* = 0.705). Higher mean scores in negative affect were found in RNs (β = 10.9, *p* = 0.002), followed by FPs (β = 7.9, *p* = 0.050) and CHWs (β = 6.3, *p* = 0.004). CHWs showed the lowest satisfaction with life (β = −7.9, *p* < 0.001), whilst RNs and FPs showed no significant differences compared to NAs (β = 0.4, p = 0.899 and β = 0.7, *p* = 0.848, respectively).

PHPs who were in the job for 1 year or more, compared to 6 months or less, showed significant differences in subjective well-being, demonstrating lower positive affect (β = −9.0, *p* < 0.001), increased negative affect (β = 5.3, *p* = 0.020), and decreased satisfaction with life (β = −5.0, *p* = 0.023). They also showed higher differences in stress (β = 4.8, *p* = 0.08).

### Associations between mindfulness, stress, and well-being

**Fig. 1:**
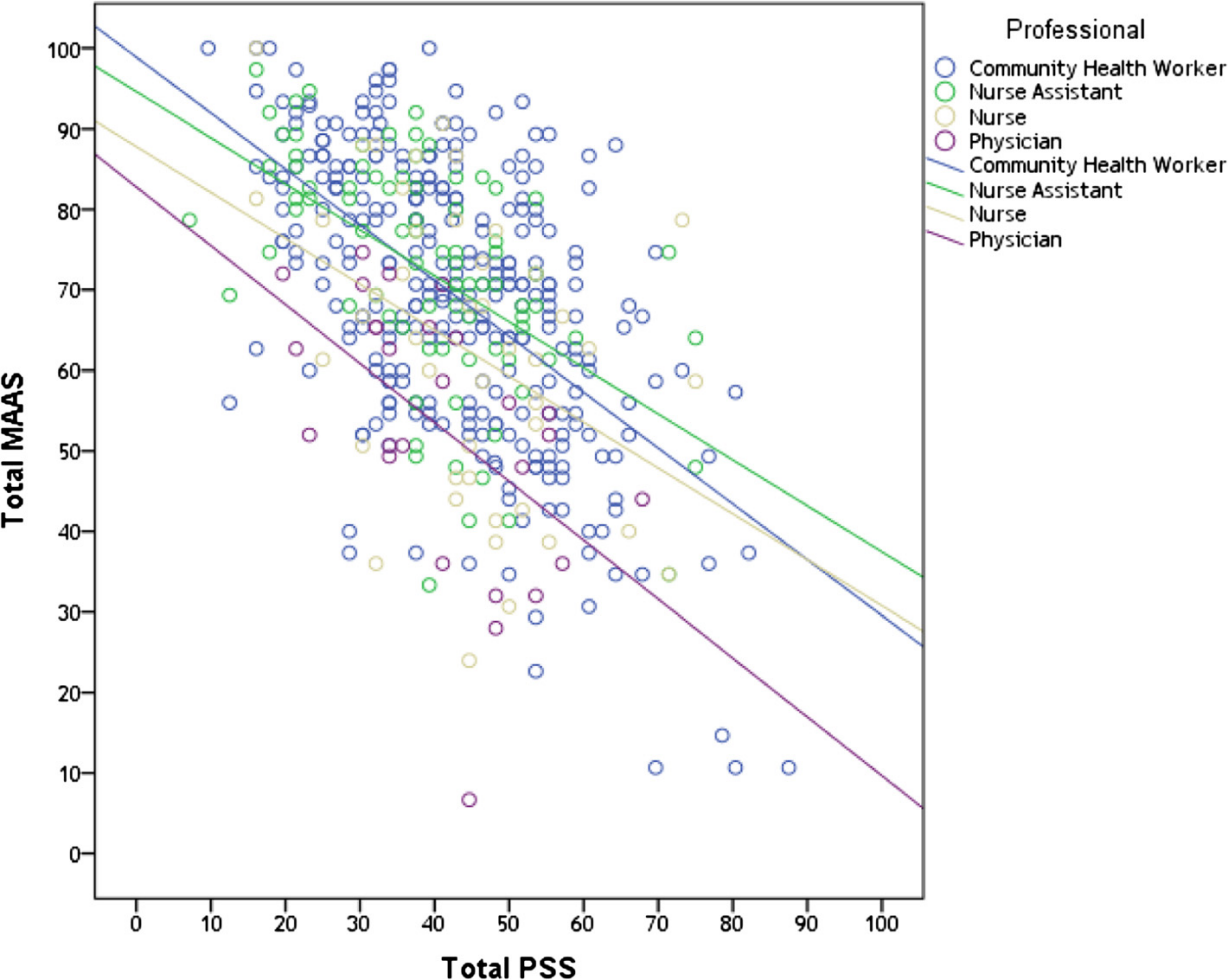
Scatterplot of PSS and MAAS scores by health professional category

Pearson’s coefficient (Table 4) indicated a strong correlation between MAAS and PSS (*r* = −0.541; *p* < 0.001). MAAS was also convergent with SWS_PA (*r* = 0.451; *p* < 0.001) and SWS_SL (*r* = 0.380; *p* < 0.001), and divergent with SWS_NA (*r* = −0.468; *p* < 0.001). We also found that professionals paying attention to and aware of the present moment tended to be less stressed, with different professional categories showing similar correlations at hierarchical levels [Fig. 1].

**Table 4:** Pearson’s correlations of the MAAS, PSS, and SWS adjusted by professional category and length of service

		PSS	SWS_PA	SWS_NA	SWS_SL
SWS_PA	Correlation	−0.589	1		
	p	<0.001	─		
SWS_NA	Correlation	0.656	−0.480	1	
	p	<0.001	<0.001	─	
SWS_LS	Correlation	−0.566	0.568	−0.568	1
	p	<0.001	<0.001	<0.001	─
MAAS	Correlation	−0.541	0.451	−0.468	0.380
	p	<0.001	<0.001	<0.001	<0.001

## Discussion

This study looked at the relationship of perceived stress, subjective well-being, and mindfulness across different PHP categories. The professional category distribution and socio-demographic features of participants were expected in PHP services in Brazil [11]. When focusing on the levels of perceived stress and subjective well-being, our results showed higher levels of stress predominantly in RNs and CHWs. This is congruent to previous research showing that in the FHS, these categories are at most risk of developing burnout [10]. Both RNs and CHWs also displayed low levels of subjective well-being, and showed the lowest levels of positive affect and significant negative affect, which reinforces evidence showing that both categories are under strain. RNs play a significant role in the FHS health model, with high levels of responsibility and workload: planning, managing, and coordinating services, as well as holding supervisory roles and delivering further training for CHWs and NAs. Furthermore, during data collection we noticed that many community-oriented primary care units had an absence of FPs, increasing the workload under the responsibility of RNs. On the other hand, the CHW’s role includes living and working directly within the community (normally associated with complex needs, violence, poverty, infectious disease). In addition, the literature on CHWs demonstrates this type of professional to be the most vulnerable to burnout due to the propensity of becoming enmeshed with the suffering showed by service users [9, 19]; which may explains our results of CHWs displaying significantly low SW_ satisfaction with life.

Broadening our discussion of well-being and stress on PHPs, the UK National Health Services recognized the need for service managers to focus on staff well-being, through the implementation of interventions aiming at a good relationship with the team, good management, and organizational structure [20]. Similarly, the literature on PHP teams working in the FHS in Brazil, reports power struggles, conflicts of opinion, and lack of service organization as risk factors contributing to high levels of stress [11, 21]. As such, our results show that compared to being in the job for under 6 months, being in the job for over one year meant higher PS and negative affect, as well as lower positive affect and satisfaction with life. An interesting line of research could be how these feelings of discomfort might evolve into the different burnout types over time, as a potential curvilinear relationship between time in job and burnout is expected [22].

With regard to the Brazilian version of MAAS on this population, the scale had good correlations with relational constructs, which is consistent with previous research [16]. The internal consistency of the scale was within the average range (0.80 to 0.90), proposed by the author of the original MAAS [6]. As expected, MAAS mindfulness was positively correlated to SW_Satisfaction with life and SW_Positive Affect, and was negatively correlated to PS and SW_Negative affect. The construct of MAAS mindfulness relates to the ability of one performing with present moment attention and awareness [6]. Our findings that RNs and FPs show the lowest levels of mindfulness are congruent with previous research linking levels of stress to poor attention performance, deficient communication, work disorganization, and lack of productivity in PHPs working in the FHS [10].

Mindfulness also refers to openness to experience, and mindfulness-based programs have been demonstrated to increase the clarity of values and ability to withstand exposure [23], to one’s capacity to sustain openness to unpleasant/pleasant dynamics, without becoming cut off from awareness of the present moment. This, in theory, leads to a more real and healthier experience, based on acceptance of reality rather than its suppression [24]. The concept of acceptance involving mindfulness, as a prerequisite to the relationship between health professionals and patients is emphasized by Schmidt [24], linking acceptance to empathy in his statement: “accepting, warm-hearted relationship with self is primary to any healing intention” ([24]; p. S7), as the capacity of one connecting with one’s own suffering, to then connecting with the suffering of others. Empathy was found to be a mediator between mindfulness and PS, showing dispositional mindfulness to have an indirect effect on PS mediated through the regulation of emotion and the ability of critical care personnel to use emotions (e.g., empathy) [25]. This could explain the high mean values of MAAS mindfulness in CHWs working for the Brazilian health service. This category, although not classified as working with critical care, is considered to be working under the highest levels of stress as a result of “being part of” and “direct work with” a vulnerable community. And empathy is evident through statements reported from qualitative research studying this population: *“I keep looking at that big queue of patients and I keep thinking - What if it was me there…”* ([26]; p.1309).

However, taking into consideration the challenges of providing service and the poor working conditions reported by research on PHPs working in the FHS, it was striking that mean values of SW_Positive Affect and SW_ Satisfaction with Life, exceeded mean values of Perceived Stress and SW _Negative Affect.

One possibility may relate to experiential avoidance, which is a psychological process in which individuals block situations and stimuli for self-protection from exposure to high levels of stress [27]. This resonates with Marqui et al. [11], who show the tendency of PHPs to assume a passive posture in relation to work difficulties as well as experiential blocking [11]. This leads to the reflection that in our study there may be a replication of this phenomenon, and as such, reinforce the lack of perceptive awareness in this population.

Another possibility is that answering instruments in the workplace may have predisposed subjects to social desirability biases. Other limitations include the cross-sectional design not revealing the nature and causal direction of the relationships found; the disparity in sample sizes of some categories, such as the large numbers of CHWs (65.8 %) and small numbers of FPs (6.0 %), due to the structure of the FHS; research based solely on self-report questionnaires and depending on motivation and the correct understanding of questions. For instance, the high (above average) levels of MAAS mindfulness in our study could therefore express misunderstanding of the questionnaire by CHWs, coupled with the high representation of this category in our sample. Further restrictions of sampling include data collected only in one region of Sao Paulo and the exclusion of PHPs on leave and off work, for instance, due to stress-related health conditions.

Despite the aforementioned, the relatively large sample of the study provides further support for the relationship between trait mindfulness, subjective well-being, and perceived stress. To our knowledge, this was the first study involving those outcome variables across different categories of PHPs, adjusted for time working in the same role. One consideration for future research is to use additional questionnaires and alternative data-collection methods to support the findings. For instance, cortisol in saliva for the investigation into mindfulness and stress [6, 28], and event-related brain potential (ERP) markers as measures for mindfulness [29] and subjective well-being [30]. Particularly for this population, future research could focus on investigating the acceptability of mindfulness-based interventions across different PHP categories, to provide to service managers with further understanding on how mindfulness can be best applied into the health service for staff self-care.

## Conclusions

This was the first study to investigate mindfulness, subjective well-being, and stress on Brazilian PHPs. As it particularly highlights the dynamics involving these associations across different professional health categories, the current work gives us a picture of how these relationships and specific vulnerabilities may affect PHPs differently. The findings potentially encourage reflections around practitioners’ level of awareness affecting their health, well-being, and work. The study explores a variety of elements that can support the development of mindfulness interventions for stress prevention, staff well-being, and improvement of services as a whole.
